# Patterns and Determinants of Habitat Occupancy by the Asian Elephant in the Western Ghats of Karnataka, India

**DOI:** 10.1371/journal.pone.0133233

**Published:** 2015-07-24

**Authors:** Devcharan Jathanna, K. Ullas Karanth, N. Samba Kumar, Krithi K. Karanth, Varun R. Goswami

**Affiliations:** 1 Centre for Wildlife Studies, Bangalore, India; 2 Wildlife Conservation Society, India Program, Bangalore, India; 3 Wildlife Conservation Society, Global Conservation Program, Bronx, New York, United States of America; 4 Nicholas School of Environment, Duke University, Durham, North Carolina, United States of America; University of Florida, UNITED STATES

## Abstract

Understanding species distribution patterns has direct ramifications for the conservation of endangered species, such as the Asian elephant *Elephas maximus*. However, reliable assessment of elephant distribution is handicapped by factors such as the large spatial scales of field studies, survey expertise required, the paucity of analytical approaches that explicitly account for confounding observation processes such as imperfect and variable detectability, unequal sampling probability and spatial dependence among animal detections. We addressed these problems by carrying out ‘detection—non-detection’ surveys of elephant signs across a *c*. 38,000-km^2^ landscape in the Western Ghats of Karnataka, India. We analyzed the resulting sign encounter data using a recently developed modeling approach that explicitly addresses variable detectability across space and spatially dependent non-closure of occupancy, across sampling replicates. We estimated overall occupancy, a parameter useful to monitoring elephant populations, and examined key ecological and anthropogenic drivers of elephant presence. Our results showed elephants occupied 13,483 km^2^ (*SE* = 847 km^2^) corresponding to 64% of the available 21,167 km^2^ of elephant habitat in the study landscape, a useful baseline to monitor future changes. Replicate-level detection probability ranged between 0.56 and 0.88, and ignoring it would have underestimated elephant distribution by 2116 km^2^ or 16%. We found that anthropogenic factors predominated over natural habitat attributes in determining elephant occupancy, underscoring the conservation need to regulate them. Human disturbances affected elephant habitat occupancy as well as site-level detectability. Rainfall is not an important limiting factor in this relatively humid bioclimate. Finally, we discuss cost-effective monitoring of Asian elephant populations and the specific spatial scales at which different population parameters can be estimated. We emphasize the need to model the observation and sampling processes that often obscure the ecological process of interest, in this case relationship between elephants to their habitat.

## Introduction

The Asian elephant (*Elephas maximus* Linnaeus, 1758), despite being a species of immense cultural, ecological and conservation value, continues to be threatened by the loss, fragmentation and degradation of habitat, conflict with humans and poaching for ivory [1–3]. Even within India––a country that harbors nearly 60% of the current global wild population––much of the species’ former range has been lost to agricultural expansion, human settlements and developmental projects in the past 2–3 centuries [2,4].Consequently, elephants in India persist today in small, insular populations, restricted largely to protected areas [5,6] within fragmented landscapes. Therefore, understanding factors that underlie elephant spatial distribution patterns is critical to prioritize habitat conservation, identify threats that limit elephant presence and to inform management actions to secure these populations over time. Notwithstanding this need, factors that drive distribution patterns and the actual area occupied by elephants in India are poorly studied and largely unknown. In contrast, several studies have addressed the question of what determines the distribution of African elephants (e.g. [7–29]). The understanding thus gained on the ecology and conservation status of elephants in Africa has long informed their intensive management [30].Not only are such data scarce in the case of Asian elephants (but see[31–38]), what is known about their distribution patterns has been generated using varied field methods (dung encounter rates, direct observation, telemetry-based locations) and diverse analytical approaches (comparisons of summary statistics, correlations, multivariate analyses, presence-only modeling), none of which accounts for observation processes such as sample selection bias, imperfect and variable detectability[39–41]. Because detectability can vary with habitat, ignoring it could seriously bias inferred occupancy-habitat relationships[41–45].A recent study on Asian elephant occupancy that did account for detectability [46] found that estimated detectability ranged from 0.08 to 0.83, depending on covariate values and intensity of use by elephants, indicating that detection is both imperfect and highly variable. Moreover, most surveys of Asian elephant distribution were not conducted at appropriate spatial scales, given their wide-ranging behavior (see[47,48]for estimates of Asian elephant home ranges).

At the local scale, past efforts to monitor Asian elephant populations have focused on estimation of population size and density. However, regional, national and range-wide estimates are based on indefensible extrapolation of such local-scale (i.e. individual reserve) estimates that vary widely in their reliability[4]. Blake and Hedges[4] justifiably worry that beyond locations and relative abundances of some of these elephant populations, we know very little. Not only have unreliable field and analytical methods been applied to estimate elephant numbers, there is also a critical mismatch between the population parameter being estimated (i.e. abundance or density) and the spatial scale of such estimation effort. While abundance and density can be reliably estimated at smaller spatial scales (e.g. individual reserves) using appropriate methods[40,49],estimates of abundance or density at larger scales tend to be unreliable (see[4]).

The appropriate metric for landscape level studies in wide-ranging species such as elephants is the estimation of habitat occupancy: the probability (Pr) that a species is present within a site(see[44,50]). Formal occupancy modeling explicitly accounts for imperfect detectability and other observation processes, which, if ignored, could lead to biased inferences of occupancy, habitat relationships and temporal changes in these. The approach also permits modeling of ecological and anthropogenic effects on detection probabilities as well as habitat occupancy (or both). This removes biases from heterogeneity among sites, in either detectability or occupancy, as well the confounding of true patterns in occupancy with detectability and sampling probability[41,44]. Consequently, rigorous occupancy modeling is rapidly emerging as a method of choice. It has been successfully used on other wide-ranging species, such as the tiger *Panthera tigris* [51].

In this study, we used habitat occupancy sampling of Asian elephants over a large and diverse landscape in the Western Ghats of Karnataka State in India. We employed a modeling approach recently developed by Hines et al. [52] that relies on spatial rather than temporal replication and uses surveys of animal signs, which has been successfully used to investigate the distribution of tigers [51] and dhole *Cuon alpinus* [53] based on the same survey effort. Our estimates provide a useful baseline to monitor future changes in spatial distribution of the largest wild population of Asian elephants. We also elucidate the role of key ecological and anthropogenic covariates as determinants of elephant habitat occupancy. We demonstrate how this approach can improve future management and monitoring of Asian elephants.

## Materials and Methods

This study only involved non-invasive surveys of elephant signs, for which the Karnataka Forest Department provided field research permits.

### Study area

**Fig 1:**
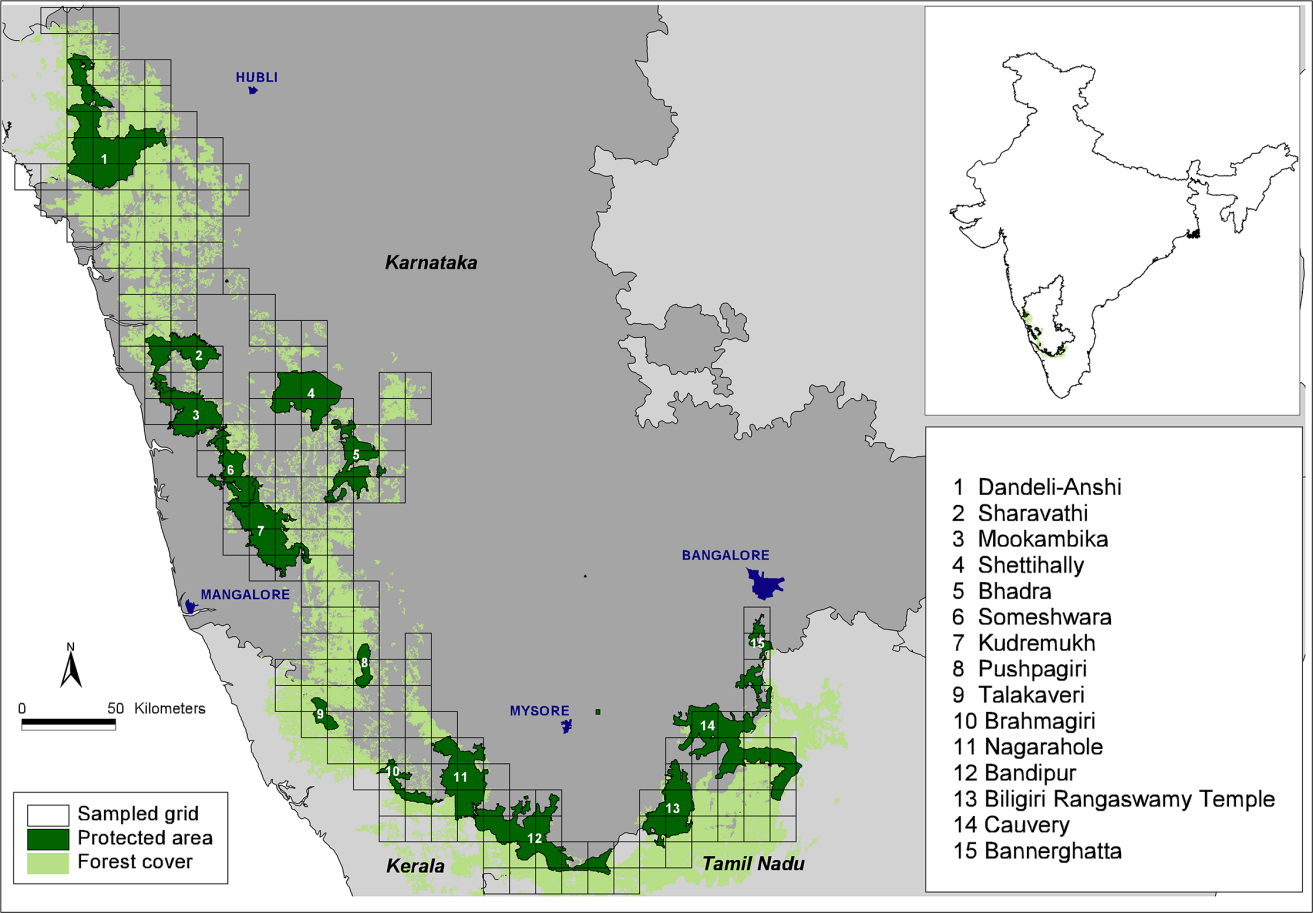
Map of our study landscape showing forest cover, protected areas and sampled grids. Inset shows location of the landscape within India.

We conducted our surveys across a c. 38,000-km^2^ landscape (Fig 1), with our sampling frame defined by the presence of natural vegetation types including forest (evergreen, moist deciduous, dry deciduous, thorn scrub), tree plantations (e.g. *Tectona grandis*, *Acacia auriculiformis*, *Eucalyptus* spp. *Casuarina equisetifolia*, among others), tree savanna, shrub savanna, grassland (in our landscape, primarily montane grasslands) and uncultivated revenue department or private lands. Thus, other than a few areas where small numbers of itinerant and conflict-prone elephants subsist in heavily human-dominated areas (e.g. in Tumkur and Hassan districts) and which have been identified for removal [54], our field surveys covered the entire distributional range of elephants in the state of Karnataka. The surveyed areas encompassed the full gradients in rainfall, land cover and intensity of human use.

The altitude in the study area peaks at 1927m, with the terrain abruptly rising from the coastal plains and then descending from the ridge of the mountains eastwards, intergrading into the Deccan Plateau. The mean annual rainfall declines from well over 5000 mm in the coastal plains and crest of the Ghats to ~600 mm eastwards [55]. Western slopes of the Ghats support wet evergreen rainforests and montane *shola*-grassland habitats; semi-evergreen and moist and dry deciduous forest types dominate areas eastward, where rainfall declines progressively. These blocks of natural forests are interspersed with or abut areas under varied forms of agriculture, horticulture and plantations as well as human settlements. We defined elephant habitat as comprising all the natural vegetation types described above, which together cover 21,167 km^2^ of the study landscape [51], and support some of the largest populations of elephants as well as other threatened large mammal species typical of the region [56,57].

The landscape includes 14 wildlife reserves that cover an area of 5,500 km^2^ and lie in a human-dominated matrix populated by >10.2 million people. The elephant habitats are subject to threats from illegal hunting, livestock grazing and forest biomass extraction as well as pressures from developmental projects and industrial growth [51]. For detailed accounts of the study area see [51,56,58,59].

### Occupancy survey design

Our goal was to estimate the proportion of the available potential habitat occupied by elephants. Thus defined, defined, ‘habitat occupancy’ corresponds to second-order habitat selection (selection of home ranges within the species’ distributional range) under the hierarchical framework proposed by Johnson [60]. Our surveys were specifically designed for tigers [51] therefore the study design, field survey protocols and analytical approach used were planned considering tiger biology. We carefully reviewed these elements of our study in the context of surveying elephant occupancy to ensure that the modeling approach did indeed describe our observation process (variably imperfect detection of signs on successive segments along trails within specified grid cells) overlaid on the ecological process of interest (true elephant distribution, determined by the values of one or more covariates). We also carefully reviewed the literature on elephant home range size to confirm that the same survey design would be appropriate for Asian elephants. Sukumar [33] estimated home ranges of two elephant families at 105 km^2^ and 115 km^2^, based on repeated observations of recognizable individuals. Further, Fernando et al.[47] reviewed a large number of Asian elephant home ranges estimates and the 18 estimates of elephant home ranges in south India in their Table 1 average to 275.8 km^2^ (minimum: 105 km^2^ [33]; maximum: 800 km^2^ [61]). When considering that a) these studies were conducted over periods ranging from nine months to nearly six years, and also b) what is of relevance to our survey design is ranging patterns over the dry season (October-May) each year, we are confident that our definition of sites as grid cells of size 188 km^2^each (Fig 1) is appropriate. Because our grid cells are larger than expected mean elephant home range sizes over the dry season, we are also confident that a) our estimates of *ψ* pertain to true pr(occupancy) rather than pr(use|occupancy) and that b) spatial autocorrelation in elephant occupancy among grid cells is minimized to the extent possible. The grid cells also coincided with graticules on the 1:50,000 topographic maps, facilitating planning of field surveys. Field surveys were not carried out in grid cells with natural habitat fragments totaling <10km^2^ since elephants and other large-ranging mammals were unlikely to occupy these cells. Of the 232 grid cells covering the overall landscape, we excluded 27 cells using this forest cover-based exclusion criterion, yielding 205 cells (sites) to be surveyed.

**Table 1:** Description of covariates used to model variation in detectability and *ψ* across sites.

Covariate		Expected effects on elephant occupancy	Expected effects on elephant detectability	Supporting citation(s)
PropFOR	Proportion of grid cell covered by forest, computed prior to field surveys from forest cover layers.	Positive effect, as increasing forest cover is expected to be correlated with lower levels of various forms of human disturbance.	Positive effect, as elephant abundance is expected to be lower at low forest cover due to multiple human disturbances.	Asian: [33, 38, 46, 77]; African forest: [8, 11, 16]; African savannah:[10, 19, 27]
LVS	Proportion of 1-km replicates containing livestock sign, as measured during field surveys	Negative relationship, both directly (through competition for forage in the dry season) and as a surrogate for other, correlated human disturbances such as hunting, biomass extraction and fragmentation.	Negative effect by lowering elephant abundance due to direct competition for forage and indirectly through other, correlated human disturbances.	Asian: [33, 38, 46, 77]; African forest: [8, 11, 16]; African savannah:[10, 19, 27]
MeanRAIN	Mean annual rainfall derived from long term monthly averages at 1-km resolution from the WorldClim database[55]. Annual means computed by pixel and averaged over each 188 km^2^ grid cell using ArcGIS 10.0. Scaled prior to analysis by subtracting minimum and multiplying by 0.001, to range from 0 to 5.25	Positive effect, especially in the drier parts of our landscape, by influencing surface water availability and indirectly by determining vegetation type and productivity.	—	Asian:[32]; African savannah:[9, 12, 15, 17–22, 24, 27–29]
MeanNDVI	Normalized Difference Vegetation Index (NDVI) at 1-km resolution from the MODIS database[97]. Image from dry season (18^th^ Feb 2006) for maximum contrast between vegetation types. Averaged over forest area pixels within each grid cell using the Spatial Analyst extension in ArcGIS 10.0.	A measure of vegetation productivity, expected to have a strong positive effect in very dry to dry habitats through increased forage quantity. However, in our study area, NDVI expected to have an overall positive effect, but one that declines at high levels of vegetation productivity (see below). Varies by vegetation type.	Overall positive effect by increasing elephant abundance.	Asian:[31, 33–35, 37, 38]; African forest: [11]; African savannah: [7, 9, 12, 15, 17, 19, 21, 23, 25, 26, 29]
NDVI2	Squared NDVI values for use in models where elephant occupancy is a nonlinear (quadratic) function of NDVI	*Ψ* expected to be highest at intermediate values of NDVI, corresponding to dry and moist deciduous forests. Occupancy expected to be low at low NDVI values due to limited forage and surface water (as in African savannah elephants) and low also at high NDVI values (evergreen forests) where much of the biomass tends to be in the canopy and where plants invest in higher levels of secondary compounds for the more permanent foliage[80], thus reducing available forage.	—	Asian: [33]; African savannah:[7, 9, 12, 21]
CV(NDVI)	Coefficient of variation of NDVI across pixels within forests within each grid cell. Calculated using the Spatial Analyst extension in ArcGIS 10.0	An index of vegetation heterogeneity in forest areas within each grid cell.	—	Asian: [36]; African savannah:[13, 14, 18, 25]

Because of low elephant densities, low sighting probabilities and logistical factors, rather than sightings, we chose to survey and detect elephant presence via signs, primarily fresh dung [40]. Although direct sightings, tracks and other evidences were also recorded, we based our analyses on fresh dung, since other signs have very different times of persistence (direct sightings are instantaneous), affecting both detectability and occupancy. Because of logistical practicality (see[51]) we chose spatial [62] over temporal replication, to estimate detectability of elephant signs. To address the potential problem of spatially autocorrelated non-closure of occupancy state along such spatial replicates, we used the recently developed occupancy model of Hines et al. [52], as more fully described below.

The survey effort (distance walked) invested within each grid cell was set proportional to the extent of elephant habitat within the cell. Elephant habitat was defined based on forest cover, classified using a rule-based eco-climatic vegetation classification [56,63]. The sampling effort was set at 40 km for cells with 100% forest cover (188 km^2^), and, decreased in proportion to extent of available habitat. Field surveys ensured adequate spatial coverage of cells but surveyors’ subjective route choice was constrained by dividing the grid cell into 16 sub-cells and ensuring that the field teams traversed one sub-cell randomly selected prior to the surveys [51].

### Field surveys

Prior to the surveys, 6 permanent staff and about 12 long-term volunteers were trained by one of the authors (NSK). We conducted a pilot training survey to familiarize the teams with field protocols. Four core teams, each led by a regional coordinator, covered different parts of the landscape. Field research teams surveyed forest trails typically used by elephants during the dry seasons (October-May) of 2005–2006 and 2006–2007. Dry season surveys ensured that dung persistence was consistent, reducing heterogeneity in detection probability induced by rainfall variation [64,65]. In addition to detection or non-detection of elephant dung, habitat covariate information was also recorded. The data were collected for each trail segment of 100 m, but were subsequently aggregated to 1-km spatial replicates (see [51]). To limit the number of parameters to be estimated, covariates of detectability were estimated only at the site level. The data were used to construct elephant sign detection histories for each cell, ‘1’ representing detection and ‘0’ non-detection [44,50]. Thus detections along trails were used to assess elephant occupancy at the site (= grid cell) level, and not at the level of each trail or trail segment. The number of replicates surveyed per cell, which depended on the proportion of habitat in it, ranged from 4–42 (also see[51]).

### Modeling elephant occupancy

#### Accounting and testing for spatial dependence in our data

An important assumption of the MacKenzie et al. [50] model is that occupancy status does not change among replicates within each site. Our surveys, using spatial replication may not always have met this assumption, because elephant presence in one part (or replicate) within a cell (site) did not necessarily mean that other parts (or replicates) within that cell were also occupied. As shown by Kendall [66] and MacKenzie et al. [44], if such within-site changes in occupancy were to occur completely randomly, then replicate-level detectability is the product of the Pr(species is present in site *and* replicate) and Pr(species is detected | species is present in site *and* replicate), while the interpretation of Pr(site is occupied) changes to Pr(site is used). However, if between-replicate changes are non-random (e.g. Markovian, as when replicates are sampled sequentially along a trail rather than randomly from within a cell), detectability cannot be decomposed as above, and estimates of both detectability and occupancy will be biased. MacKenzie et al. [44] and Kendall & White [62] recommend sampling of spatial replicates randomly with replacement to ensure changes in occupancy within a cell are completely random. However, such an approach is logistically impractical to implement in field surveys of elephants. For this reason our elephant sign survey data were generated from sequential sampling of successive spatial replicates along trails walked by teams. To fully account for this factor, we chose to model occupancy using a recent approach developed by Hines et al. [52]. This model explicitly accounts for spatial dependence in elephant presence at the replicate level as a first-order spatial Markov process. Parameters estimated from this model are *ψ* = Pr(site is occupied by elephants), *θ* = Pr(elephants present on replicate | site is occupied and elephants absent on previous replicate), *θ*′ = Pr(elephants present on replicate | site is occupied and elephants present on previous replicate) and *p* = Pr(detection at a replicate | elephants present both in the site and in the replicate). Because the first replicate could be located anywhere within each cell, we calculated probability of replicate-level presence for the first replicate *θ*_0_ as the weighted (by *ψ)* average of *θ* and *θ*′.

This practically useful occupancy modeling approach was earlier developed specifically for surveys of tigers in our study area (see[51,52] for details). Because elephants do not use forest roads and trails as intensively as tigers do, we were unsure if there was substantial spatial dependence in elephant presence among successive replicates. Therefore, we initially tested for possible existence of spatial dependence in replicate-level elephant presence by comparing the fits of the standard MacKenzie et al. [50] model and the Hines et al. [52] model to our survey data based on AIC values [67], and also by comparing the estimated values of *θ* and *θ*′. We did not assess fit of the models to the data [68] as there is currently no way to assess fit under the Hines et al. [52] modeling approach.

#### Modeling covariate effects on detectability and occupancy

Another major objective was to assess the role of ecological and anthropogenic factors that possibly determined elephant occupancy in our landscape. This involved realistically modeling variation in sign detectability also, so that patterns in detectability were not wrongly attributed to patterns in occupancy. The plausible covariates that influenced detectability and/or elephant occupancy included data collected in the field surveys as well as remotely sensed data. Modeling of such covariates is important to ensure there is no un-modeled variation in Pr[occupancy] or detectability among cells [44,50]. More importantly, comparing likely covariate models enables examination of methodological (what influenced detectability) as well as important ecological or conservation questions (what determines elephant spatial distribution/occupancy). Based on our empirical experience, and, previous studies of Asian and African elephants (*Loxodonta cyclotis* Matschie, 1900 and *L*. *africana* Blumenbach, 1797), we identified covariates that we expected to influence probability of habitat occupancy or detectability, or both, for elephants in our study area (Table 1). We note that some of these covariates were expected to potentially influence both *ψ* as well as *p*. Influences on detectability included both direct (e.g. rainfall, which determines dung persistence) as well as indirect effects (covariates that affect elephant abundance in the site, which then affects species-level detectability). We first assessed collinearity between the potential predictors using scatter plots and Pearson’s correlation coefficients. We did not include in the same model covariates that were substantially correlated (Pearson’s |*r|*> 0.7; [69]). To limit the number of models in our candidate set, we first assessed the role of four potential covariates of *p* (see Table 1) while holding the covariate structure for *ψ* constant, following Karanth et al. [51] (see [70] for a discussion of such two-step analyses). Because we effectively conditioned on the covariate structure for occupancy while assessing covariates for detectability, we chose a moderately parameterized form for modeling occupancy in this step (Table 2). Based on AIC values of the different models of detectability (Table 2), we then fixed the covariate structure for *p* and assessed the influence of our covariates on *ψ*, also based on AIC values (Table 3).

**Table 2:** Model selection results: Covariate effects in determining detectability ${\hat{p}}_{t}$ on 1-km-long spatial replicates, based on the Hines et al. (2010) modeling approach. No. of sites = 205. Please see Table 1 for descriptions of covariates.

Model	AIC	Δ AIC	AIC weight	Model likelihood	No. parameters	Deviance
psi(PropFor+NDVI+LVS),thta0,thta1,p(LVS+PropFor)	2890.8	0.00	0.4953	1	9	2872.8
psi(PropFor+NDVI+LVS),thta0,thta1,p(LVS+PropFor+AnnuRain)	2892.18	1.38	0.2485	0.5016	10	2872.18
psi(PropFor+NDVI+LVS),thta0,thta1,p(LVS+PropFor+NDVI)	2892.79	1.99	0.1831	0.3697	10	2872.79
psi(PropFor+NDVI+LVS),thta0,thta1,p(LVS)	2896.01	5.21	0.0366	0.0739	8	2880.01
psi(PropFor+NDVI+LVS),thta0,thta1,p(LVS+AnnuRain)	2897.11	6.31	0.0211	0.0426	9	2879.11
psi(PropFor+NDVI+LVS),thta0,thta1,p(LVS+NDVI)	2897.75	6.95	0.0153	0.031	9	2879.75
psi(PropFor+NDVI+LVS),thta0,thta1,p(.)	2932.15	41.35	0	0	7	2918.15
psi(PropFor+NDVI+LVS),thta0,thta1,p(AnnRain)	2933.19	42.39	0	0	8	2917.19
psi(PropFor+NDVI+LVS),thta0,thta1,p(NDVI)	2933.68	42.88	0	0	8	2917.68
psi(PropFor+NDVI+LVS),thta0,thta1,p(AnnuRain+PropFor)	2934.28	43.48	0	0	9	2916.28
psi(PropFor+NDVI+LVS),thta0,thta1,p(NDVI+PropFor)	2934.71	43.91	0	0	9	2916.71
psi(PropFor+NDVI+LVS),thta0,thta1,p(PropFor)	2938.83	48.03	0	0	8	2922.83

**Table 3:** Model selection results: Covariate effects in determining probability of elephant occupancy in our study landscape, based on the Hines et al. (2010) modeling approach. No. of sites = 205. Please see Table 1 for descriptions of covariates.

Model	AIC	Δ AIC	AIC weight	Model likelihood	No. parameters	Deviance
psi(LVS*NDVI),thta0,thta1,p(LVS+PropFor)	2849.53	0.00	0.6622	1	9	2831.53
psi(LVS+PropFor+AnnuRain),thta0,thta1,p(LVS+PropFor)	2852.56	3.03	0.1456	0.2198	9	2834.56
psi(LVS+AnnuRain),thta0,thta1,p(LVS+PropFor)	2852.63	3.10	0.1405	0.2122	8	2836.63
psi(LVS*AnnuRain),thta0,thta1,p(LVS+PropFor)	2854.63	5.10	0.0517	0.0781	9	2836.63
psi(LVS+NDVI),thta0,thta1,p(LVS+PropFor)	2889.23	39.70	0	0	8	2873.23
psi(NDVI+NDVISQ+LVS),thta0,thta1,p(LVS+PropFor)	2889.85	40.32	0	0	9	2871.85
psi(LVS+PropFor+NDVI),thta0,thta1,p(LVS+PropFor)	2890.8	41.27	0	0	9	2872.8
psi(PropFor*AnnuRain),thta0,thta1,p(LVS+PropFor)	2899.57	50.04	0	0	9	2881.57
psi(PropFor+AnnuRain),thta0,thta1,p(LVS+PropFor)	2899.73	50.20	0	0	8	2883.73
psi(LVS),thta0,thta1,p(LVS+PropFor)	2901.53	52.00	0	0	7	2887.53
psi(LVS+CVNDVI),thta0,thta1,p(LVS+PropFor)	2903.31	53.78	0	0	8	2887.31
psi(LVS+PropFor),thta0,thta1,p(LVS+PropFor)	2903.45	53.92	0	0	8	2887.45
psi(LVS*PropFor),thta0,thta1,p(LVS+PropFor)	2905.44	55.91	0	0	9	2887.44
psi(AnnuRain),thta0,thta1,p(LVS+PropFor)	2906.13	56.60	0	0	7	2892.13
psi(NDVI+CVNDVI),thta0,thta1,p(LVS+PropFor)	2931.58	82.05	0	0	8	2915.58
psi(NDVI+PropFor),thta0,thta1,p(LVS+PropFor)	2931.71	82.18	0	0	8	2915.71
psi(NDVI+NDVISQ+PropFor),thta0,thta1,p(LVS+PropFor)	2933.15	83.62	0	0	9	2915.15
psi(NDVI*PropFor),thta0,thta1,p(LVS+PropFor)	2933.53	84.00	0	0	9	2915.53
psi(NDVI),thta0,thta1,p(LVS+PropFor)	2933.56	84.03	0	0	7	2919.56
psi(NDVI+NDVISQ),thta0,thta1,p(LVS+PropFor)	2935.19	85.66	0	0	8	2919.19
psi(PropFor+CVNDVI),thta0,thta1,p(LVS+PropFor)	2935.31	85.78	0	0	8	2919.31
psi(.),thta0,thta1,p(LVS+PropFor)	2935.92	86.39	0	0	6	2923.92
psi(PropFor),thta0,thta1,p(LVS+PropFor)	2936.38	86.85	0	0	7	2922.38
psi(CVNDVI),thta0,thta1,p(LVS+PropFor)	2936.92	87.39	0	0	7	2922.92

In addition to additive effects of covariates on occupancy, we also included interactions between some of the covariates where we expected the influence of one covariate on *ψ* to depend on the value of another covariate. Pettorelli et al. [71,72] show that NDVI is an excellent index of vegetation productivity and list several examples of the successful use of NDVI to understand patterns of distribution and abundance of animals. We included a quadratic term for vegetation productivity in some models because we expected probability of elephant occupancy to exhibit a non-linear, peaked response from low (thorn scrub-tree savannah-dry deciduous) through medium (moist deciduous) to highly productive (wet evergreen) habitats. All covariates were modeled using the logit link function[44,50]. Covariate values were scaled as described in Table 1. We used the software program PRESENCE v 4.1 [73] to implement all these occupancy models and to estimate relevant parameters.

#### Estimation of overall elephant occupancy in our landscape

Following Karanth et al.[51], overall occupancy across the landscape was computed by weighting each site-specific occupancy estimate by the area of forest cover in that site as a proportion of the entire 21,167 km^2^ landscape, *w*_*i*_ and summing over all 205 sites. Standard error of overall occupancy was computed as

$$S\hat{E}(\hat{\psi })=\sqrt{\sum_{i=1}^{205}w_{i}^{2}\;\text{v}\hat{\text{a}}\text{r}{\hat{\psi }}_{i}+\sum_{i=1}^{205}\sum_{\begin{array}{l} j=1\\ j\neq i \end{array}}^{205}w_{i}w_{j}\;\text{c}\hat{\text{o}}\text{v}({\hat{\psi }}_{i}{\hat{\psi }}_{j})}$$

The covariance between estimated site-specific occupancy estimates in the expression above was estimated using a parametric bootstrap [74] where the untransformed *β* parameter estimates and the associated variance-covariance matrix from the best model were used to generate 100 random deviates from a multivariate normal distribution using the R package MSBVAR [75,76]. These simulated *β* values were then used to compute 100 site-specific occupancy probabilities for each of the 205 sites using the inverse logit link function. For each pair of site-specific occupancy estimates, covariance was then computed as shown below (please see Appendix S2 in [51] for more details):

$$\text{cov}(\psi_{i},\psi_{j})=\frac{\sum_{i=1}^{100}\left(\psi_{i}-{\bar{\psi }}_{i})(\psi_{j}-{\bar{\psi }}_{j}\right)}{99}$$

## Results

### Description of the dataset

We surveyed *n* = 205 sites, covering 21,167 km^2^ of forests within our study landscape. In each site, we surveyed 4–42 1-km long spatial replicates (mean effort per site = 20.36km) depending on the amount of forest cover available in the site. A total walk effort of 4,172 km resulted in 2,712 detections of fresh elephant dung. In terms of frequencies, at least one elephant dung pile was detected in 1,230 of the 4,172 1-km replicates (0.295) and in 110 of the 205 sites (naïve occupancy = 0.537).

### Occupancy modeling

#### Spatial dependence in our data

A comparison of the MacKenzie et al.[50] and Hines et al. [52] models clearly shows that replicate-level elephant presence was spatially correlated (Δ AIC = 641.7 for the MacKenzie et al. [50] model). Additionally, the probability of replicate-level elephant presence estimated using the Hines et al.[52] model without covariates, differed substantially depending on whether elephants were present or not present on the previous replicate ($\hat{\theta }\prime [S\hat{E}]$ = 0.953 [0.008] and $\hat{\theta }[S\hat{E}]$ = 0.084 [0.016], respectively). This finding indicates strong Markovian dependence in replicate-level presence. We therefore used the Hines et al.[52] modeling approach for all subsequent occupancy analyses. As expected, detectability was negatively biased when the MacKenzie et al.[50] model was used ($\hat{p}[S\hat{E}]$ = 0.511 [0.010]) compared to the estimate from the Hines et al. [52] model (${\hat{p}}_{t}[S\hat{E}]$ = 0.737 [0.016]).

#### Effects of ecological and anthropogenic covariates on detectability and occupancy

Initial exploratory analysis revealed substantial collinearity between mean normalized difference vegetation index (NDVI) values and mean annual rainfall (Pearson’s *r* = 0.81). Therefore, these covariates were not included together in any model. As described above, we first explored covariate effects on detectability while holding the covariate structure for occupancy constant––in this step, logit occupancy was modeled as an additive function of proportion of forest cover, NDVI and livestock presence. A model where logit detectability was an additive function of livestock presence and proportion of forest cover received the most support from the data (Table 2). There was evidence to suggest that logit detectability also varied as a function of (a) livestock presence, proportion of forest cover and rainfall (ΔAIC = 1.38) and (b) livestock presence, proportion of forest cover and NDVI (ΔAIC = 1.99). Since our primary interest was in modeling covariate effects on occupancy while accounting for heterogeneity in detectability, we used the covariate structure for detectability as indicated by the minimum AIC model for all subsequent analyses. We note that the covariate structure for occupancy used in this step includes the covariates determined to be most important in determining elephant occupancy (Table 3), with the exception of rainfall, which was highly correlated with NDVI.

Based on the literature on Asian and African elephants (Table 1), we constructed a set of 24 *a priori* candidate models (Table 3) where *ψ* was determined by one or more covariates. These models included additive effects of two or more covariates (on the logit scale), interactions between covariates and in the case of NDVI, a quadratic term for non-linear responses. The model with *ψ* as a function of the interaction between livestock presence and NDVI received the most support from the data, substantially more than the next best model (additive effects of livestock presence, proportion of forest cover and annual rainfall; ΔAIC = 3.03). We therefore did not use model averaging to derive our parameter estimates. Based on the top model, probability of replicate-level presence given (a) presence on the previous replicate and (b) absence on the previous replicate were $\hat{\theta }\prime [S\hat{E}]$ = 0.962(0.007) and $\hat{\theta }[S\hat{E}]$ = 0.082(0.015), respectively. The probability of elephant presence on the first replicate was ${\hat{\theta }}_{0}[S\hat{E}]$ = 0.683(0.035). Site-wise estimates of replicate-level detectability ${\hat{p}}_{t}$ ranged from 0.56 to 0.88, depending on covariate values, while site-wise probability of occupancy $\hat{\psi }$ ranged from 0.19 to 0.98.

Akaike weights summed over all models containing each predictor ([67]) indicated that livestock presence was by far the most important predictor of elephant occupancy (summed Akaike weight ~ 1), mean NDVI (0.662) received less support, and mean annual rainfall (0.1922) and proportion of forest cover (0.1456) were far less important. Non-linear response of occupancy to NDVI and the coefficient of variation of NDVI (as a measure of habitat heterogeneity) received practically no support from the data (summed Akaike weight ~ 0).

Estimated slope parameters from the four models with ∆AIC < 10 (Table 4) showed that elephant occupancy was negatively associated with livestock presence and positively associated with proportion of forest cover, as expected. Contrary to our expectations, the main effects for mean NDVI and mean annual rainfall, as well as the interaction between livestock presence and NDVI were all negative, while the interaction term between livestock and rainfall was positive but small. The interaction term in the top model indicated that livestock presence––a collective surrogate for human disturbance in our study (see [51] for a discussion)––had the greatest negative effect on elephant occupancy in highly productive (i.e. rainforest) habitats.

**Table 4:** Estimated *β* parameter estimates for covariates determining elephant occupancy in our study landscape, from the 4 models with ΔAIC < 10. Point estimates followed by standard error (SE) in parentheses. No. of sites = 205. Please see Table 1 for descriptions of covariates.

Model	${\hat{\beta }}_{0}(S\hat{E})$	${\hat{\beta }}_{LVS}(S\hat{E})$	${\hat{\beta }}_{NDVI}(S\hat{E})$	${\hat{\beta }}_{LVS\times NDVI}(S\hat{E})$	${\hat{\beta }}_{\text{Pr}\;opFOR}(S\hat{E})$	${\hat{\beta }}_{AnnuRAIN}(S\hat{E})$	${\hat{\beta }}_{LVS\times AnnuRAIN}(S\hat{E})$
psi(LVS*NDVI),thta0,thta1,p(LVS+PropFor)	5.582 (1.655)	-2.181 (1.014)	-3.417 (1.973)	-2.831 (0.467)	—	—	—
psi(LVS+PropFor+AnnuRain),thta0,thta1,p(LVS+PropFor)	6.479 (1.311)	-6.078 (1.169)	—	—	1.338 (0.930)	-1.150 (0.200)	—
psi(LVS+AnnuRain),thta0,thta1,p(LVS+PropFor)	7.291 (1.239)	-6.352 (1.178)	—	—	—	-1.091 (0.191)	—
psi(LVS*AnnuRain),thta0,thta1,p(LVS+PropFor)	7.354 (2.163)	-6.423 (2.347)	—	—	—	-1.113 (0.664)	0.026 (0.786)

#### Overall elephant occupancy in our landscape

Dividing the number of cells in which elephants were detected by the total number of cells (205) yielded a naive (i.e. assuming detectability = 1) occupancy estimate of 0.537. However, based on site-specific occupancy estimates from the minimum AIC model, overall occupancy was estimated as $\hat{\psi }(S\hat{E})$ = 0.637 (0.04). Thus of the 21,167km^2^ of forest area within our study landscape, we estimated that 64% or 13,483 km^2^ (*SE* = 847 km^2^) was actually occupied by elephants in the Western Ghats of Karnataka. In comparison, the naïve occupancy estimate of 0.537, as derived using a traditional presence-absence analysis of our survey data, yields an estimated 11,367 km^2^ occupied by elephants, an underestimation of true occupancy by *c*. 16%.

## Discussion

### Overall occupancy and covariate effects on detectability and occupancy

Our estimate of overall occupancy (the proportion of area occupied by elephants) of 64% (~13,500km^2^) is the first such estimate based on scientifically robust methodology for this landscape. It provides a baseline against which future changes in elephant distribution can be rigorously assessed. Our survey design and analytical approaches explicitly modeled the (a) observation processes through which data were collected and (b) effects of biologically reasonable covariates on both elephant habitat occupancy and detectability of their signs. We note that although our estimates of detectability are fairly high (ranging from 0.56 to 0.88 depending on covariate values), ignoring that they are less than perfect (i.e. <1.0) can seriously bias inferred occupancy-habitat (and occupancy-disturbance) relationships by (a) yielding estimated slope parameters with biased magnitude, sometimes with reversed direction and (b) changing the inferred relative importance of covariates [42–44].

Our results indicate that human intrusion and disturbance in these forests, as measured by frequency of livestock signs, is an overwhelmingly more important influence on elephant habitat occupancy compared to ecological attributes we examined. This may indicate that elephant occupancy is likely to be negatively influenced by competition for forage, particularly in the dry season. However, in this landscape and forest management system, livestock presence is also usually strongly correlated with other forms of human disturbance such as biomass extraction, habitat degradation, habitat fragmentation and hunting [51,77]. Clearly, it is not possible to tease apart such confounding effects in a purely non-experimental study such as ours. Nevertheless, our long-term empirical experience in this landscape suggests that the strong negative influence of livestock presence on elephant occupancy likely reflects the cumulative effects of several forms of human disturbances. This is not surprising given the past distribution and recent extirpation of this generalist species from apparently suitable habitats in India [6,78]. Sukumar et al. [33] and Rood et al. [38] observed a similar influence albeit using field and analytical methods that ignored imperfect detectability. Recent studies of Asian and African elephants that did account for imperfect and variable detectability also highlight the sensitivity of elephants to human disturbance [16,46]. Further, our finding that detectability was influenced by both human disturbance and proportion of forest cover in a cell suggest that in addition to influencing occupancy, anthropogenic factors may also influence elephant abundance (which can then determine detectability of the species). Although we did not use a modeling approach which explicitly models abundance effects on speciespfxpbe2tfsrtrzsv[79] because of some strong assumptions and stringent design requirements that were unlikely to have been met in our surveys, we feel that this effect of disturbance on the species’ detectability likely arises from effects on abundance, as we had expected a priori when selecting putative predictors of detectability.

The less important effects of NDVI and rainfall were negative, contrary to results from studies in Africa. As pointed out by Borowik et al. [80] in the context of seasonal differences in forests and grasslands, the ability of NDVI to reflect ground vegetation biomass is mediated by leaf cover in shrub, lower and upper canopy layers. In evergreen forest areas, high NDVI values (due to high canopy closure) may not reflect the low forage availability to elephants at lower levels. Additionally, plants may invest in higher levels of secondary compounds [81] in the more permanent foliage of evergreen forests. Deciduous forest canopies are almost completely leafless at this time of the year (our image was from 18^th^ February 2006), while the small proportion of montane grasslands in our landscape is covered by coarse, dry grasses with low nutrient and chlorophyll content and, therefore, low NDVI values. These results are consistent with the general observation that Asian elephants occur in lower densities in primary rainforests than in dry and more open forests [2].

Unlike the African savannah, where rainfall is a strong determinant of elephant occupancy (see Table 1), most areas in our landscape are well above the annual rainfall breakpoint below which rainfall alone determines woody vegetation cover [82]. In the range of rainfall (and correlated NDVI) values found in the African savannah, elephant distribution as well as abundance has been shown to be strongly positively related to NDVI. In our landscape, the minimum long-term average annual rainfall for a grid cell (averaged across all pixels within that cell) was 630 mm, the mean was 2515 mm, and the maximum was 5883 mm. In such a wet landscape, we found that elephant distribution was negatively related to both rainfall and NDVI (strongly correlated to each other), and we believe that the preponderance of extremely wet (and dense, evergreen-canopied) areas in our landscape masks the peak in pr(occupancy) in deciduous forest areas with intermediate NDVI and rainfall values. This is supported by a recent study carried out in a purely deciduous part within our study landscape [83], which found that probability of habitat use by elephants was highest in areas which experienced the least reduction in NDVI from the end of the wet to the dry season (i.e. moist deciduous forests). However, we also believe that the strong, predictable correlation of elephant occupancy with NDVI and rainfall evident in the African savannah is more complicated in our landscape, potentially due to the overriding influence of anthropogenic disturbance.

Although our estimates of overall elephant occupancy are largely relevant and limited to monitoring and conserving elephants in our landscape (which, nevertheless, supports the largest population of Asian elephants globally), we suggest that our inferred elephant–habitat relationships are fairly generalizable across the Asian elephant’s geographical range. The evergreen and deciduous forests that comprised a majority of our sampled area are found throughout the the species’ range in south and southeast Asia. Similarly, anthropogenic disturbances abound across the elephant’s range in Asia [1]. We therefore do not expect Asian elephants elsewhere to react very differently to forested habitats, rainfall regimes and, in particular, to anthropogenic pressures. While the specific types of disturbances may vary across the species’ range, causing some differences in how elephant distributions respond to levels of human activities in other landscapes compared to ours, we believe that these differences would be quantitative rather than qualitative.

Elephants, either in Asia or in Africa, are megaherbivores, with similar physiological, thermoregulatory, dietary, osmoregulatory and other requirements/constraints. This is particularly true for the forest elephant in Africa that occurs in relatively similar habitats as the Asian elephant. We therefore suggest that our findings likely have considerable relevance to patterns of habitat selection by African forest (but possibly not African savannah) elephants. At the very least, we feel that our results have the potential to inform the selection of putative predictors of African forest elephant distribution in future studies.

### Management and conservation implications

Our findings clearly show that elephant distribution is currently limited more strongly by human disturbance compared to natural habitat characteristics we assessed. This result underscores the conservation value of limiting disturbances such as illegal hunting, cattle grazing, forest product and biomass extraction as well as habitat fragmentation arising from local and large scale economic development. Given that the landscape supports over 10.2 million people and is undergoing rapid economic growth, there is urgent need to provide more strictly protected enclaves for elephants across their distributional range in this landscape. Our study showed poor correspondence between elephant distribution and aridity of habitat types, even during the dry season. Given the presence of numerous natural and artificial sources of water in this habitat matrix, this is not surprising. Therefore, the utility of continually increasing surface water availability through engineering interventions, a standard management practice currently, is questionable.

**Fig 2:**
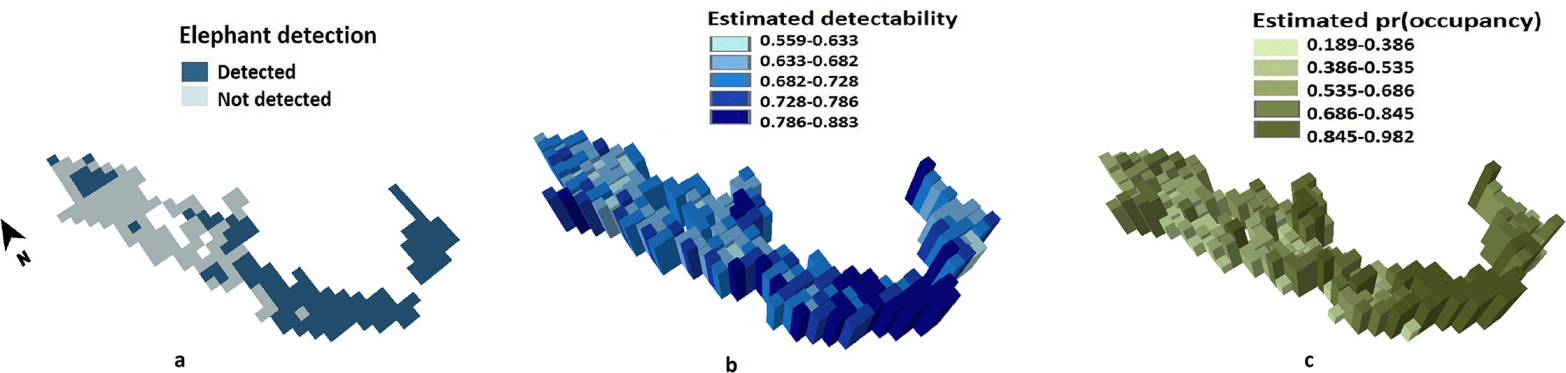
Elephant detections (a), estimated detectability (b) and estimated *ψ* (c) in our study landscape. Estimated site-specific detectabilities and *ψ* are based on the minimum AIC model.

Our occupancy analyses are also extremely useful for identifying priority sites for focused conservation of elephant populations and their habitats (Fig 2C). While this has been attempted previously (e.g. [84]), such previous analyses did not account for confounding factors such as spatially uneven sampling effort, imperfect and variable detectability and spatial dependence in detection of elephant signs, all of which can seriously affect inference and prediction. A recent study by Madhusudan et al. [54] provides a comprehensive and useful assessment of the species' distribution, relative abundance, conservation status and spatial patterns in human–elephant conflict in Karnataka, using a variety of information sources, and examines these with respect to protected area coverage, forest cover, human population density, type and extent of agriculture and extent of interface between forest and human-dominated areas. However, their assessment of elephant distribution does not account for imperfect detectability and, as also recognized by the authors, does not distinguish between occupancy and seasonal use (especially in human-dominated areas). Our sampling and modeling explicitly accounted for both these issues, and the resulting estimates and spatial predictions may therefore be considered extremely reliable. Furthermore, their assessment of relative abundance a) poorly accounts for detectability of dung piles (since they use average perpendicular distance rather than dataset-specific fitted detection functions (e.g. see Jathanna et al. [85]) to estimate dung pile density); b) is subject to biases from non-random sampling caused by the use of forest beats (area range given as 0.6–1802 km^2^) as sampling units (see Table 1, Jathanna et al. in press for a discussion) and c) is confounded with spatial variation in dung decay rates (Table 1, Jathanna et al. [85]), which we expect to be considerable, given the extremely wide range of annual precipitation across elephant habitats in Karnataka.

### Monitoring of elephant populations in India

Our study demonstrates the practical utility of occupancy sampling to the monitoring of elephant populations at larger landscape and regional spatial scales. Our approach explicitly accounts for imperfect and variable detectability of elephant signs in surveys, variation in *ψ* and spatial dependence of elephant presence on trail segments that are surveyed. We submit that the use of a modeling approach that closely describes how the data could have been generated, designing and conducting field surveys so that they match the analytical framework used, and careful modeling of observation as well as state processes (using the best available information) are critical to making inferences that can reliably and substantively inform monitoring, conservation and management of elephants as well as other wide-ranging, terrestrial vertebrate species globally. Unfortunately, the country-wide Synchronised Elephant Census (SEC) currently carried out by the Indian government relies on very different methods that attempt to directly estimate elephant population size: waterhole counts, block counts and ‘indirect’ estimates of elephant densities derived from dung counts [86, 87]. These elephant ‘numbers’ / ‘densities’ obtained in specific areas are then used to produce state-, country- and range-wide estimates of abundance through extrapolation, despite the complete lack of randomization and inadequate replication. Blake and Hedges [4] argue that such “uncritical acceptance of poor-quality data…impedes planning for effective elephant conservation.” Elephant numbers obtained from the SEC lack scientific rigor, both because of the unreliability of the methods used and the mismatch between the population parameters and the spatial scale at which they are estimated.

As noted by the Elephant Task Force Report [87] the block and waterhole count methods are not rooted in estimation theory, are subject to a number of biases, and are likely to produce misleading elephant population numbers. Although the ‘indirect method’ can be used to estimate elephant dung densities, reliably deriving elephant densities by applying dung decay and defecation rates is challenged by factors that can introduce substantial biases [88].

Abundance and density are undoubtedly more useful parameters to inform conservation and management than habitat occupancy, if and when these can be reliably estimated. At the scale of individual sites or reserves (e.g. protected areas), approaches such as line transect surveys [89] based on visual detections of elephants [90–92] or capture–recapture sampling [93] of elephants from individual identifications based on photographs or DNA [49,94–96] have proven merit for abundance or density estimation.

However, at large spatial scales such as states, regions and countries, reliable estimation of abundance or density is not just difficult, but impossible at the current time, given constraints such as finite resources and human-power, sample size requirements for reliable estimation, variable detectability (which precludes the use of indices), among other issues. Therefore, we strongly recommend that the focus at such scales be moved away from estimation of abundance or density. Rather than producing biased and dangerously misleading ‘estimates’ of state- and country-wide elephant numbers (see [4] for a critique), the emphasis should be on producing population parameters that are reliable, and useful for informing conservation at these large spatial scales. As we show in this paper, the occupancy modeling approach is ideally suited for monitoring elephants at these scales. Over time, such data can help assess changes in available habitat as well as monitor rates of colonization and extinction of sub-populations. We believe such reliable and rigorous monitoring is critical for effective long-term conservation of the Asian elephant in the face of rapidly growing economies and accelerating land use changes.

## Supporting Information

S1 File Data used to model Asian elephant occupancy in our landscape. Separate worksheets contain a) detection—non-detection data and b) covariate data.
(XLSX)
